# A Review of the Evolution of Termite Control: A Continuum of Alternatives to Termiticides in the United States with Emphasis on Efficacy Testing Requirements for Product Registration

**DOI:** 10.3390/insects13010050

**Published:** 2022-01-01

**Authors:** Faith Oi

**Affiliations:** Entomology and Nematology Department, University of Florida, 1881 Natural Area Drive, Gainesville, FL 32611, USA; foi@ufl.edu

**Keywords:** termite alternatives, termiticides, termite baits, wood treatments, physical barriers, home remedies, performance standards, building code

## Abstract

**Simple Summary:**

Termites are structurally destructive pests that can infest a consumer’s most important investment, their home. The evolution of termite control is complex, involving people’s understanding of termite biology and behavior, building construction, the use of soil termiticides, baits, wood treatments, and physical barriers, as well as regulations from different industries (pest control and building construction). Soil termiticides have been the standard method of treatment for decades, but the concern for human and environmental health has driven the development of alternatives. This article discusses the evolution of termite control methods that were alternatives to the standard of the time and the regulatory structure that provides a level of consumer protection for some products.

**Abstract:**

The global economic impact of termites is estimated to be approximately USD 40 billion annually, and subterranean termites are responsible for about 80% of the total impact. Twenty-eight species of termites have been described as invasive, and these termites are spreading, partially due to global trade, making effective control methods essential. Termite control is complex, as is the biology and behavior of this social insect group. In the U.S., termite prevention and control (with claims of structural protection) is regulated by more than one industry (pest control and building construction), and at the federal and state levels. Termite prevention has historically relied on building construction practices that do not create conducive conditions for termite infestations, but as soil termiticides developed, heavy reliance on pesticides became the standard for termite control. The concern for human and environmental health has driven the development of termite control alternatives and regulation for products claiming structural protection. Product development has also provided unprecedented opportunities to study the biology and behavior of cryptobiotic termites. Technological advances have allowed for the re-examination of questions about termite behavior. Advances in communications via social media provide unrestricted access to information, creating a conundrum for consumers and science educators alike.

## 1. Introduction

The history of termite control is one of the vetting methods that were considered to be alternatives to the standard practices of the time. As our understanding of these cryptic insects has increased, so has our ability to protect structures and their contents in more effective and environmentally sound ways. Termite control that claims structural protection in the United States is complex. It is highly regulated, fraught with the risk of litigation, and treatments can be expensive. Cost is a driving force behind property owners seeking “do-it-yourself” methods to control this structurally destructive insect. Sadly, too many consumers eventually find that the cost of repair due to inadequate termite control far exceeds the initial cost of a thorough and professional treatment.

The global economic impact of termites is estimated to be at USD 40 billion annually, and subterranean termites are responsible for about 80% of the total economic impact. Rust and Su [1] report 79 termite species that are considered serious pests and are divided among these taxonomic groupings:

- 38 species in the family Rhinotermitidae, genera: *Coptotermes*, *Reticulitermes*, *Heterotermes*, *Globitermes*, *Psammotermes*.
- 24 species in the family Termitidae, genera: *Odonototermes*, *Nasutitermes*, *Macrotermes*, *Microtermes*, *Amitermes*, *Microhodotermes*.
- 13 species in the family Kalotermitidae, genera: *Cryptotermes*, *Incisitermes*, *Glyptotermes*, *Marginitermes*.
- 3 species in the family Hodotermitidae, genus *Anacanthotermes*.
- *Mastotermes darwinensis* in the family Mastotermitidae.

Termites also are placed into common name functional groups associated with their nesting habitat, such as subterranean (e.g., *Reticulitermes*, *Coptotermes*), drywood (e.g., *Incisitermes*, *Cryptotermes*), dampwood (e.g., *Neotermes*, *Zootermopsis*), and arboreal termites (e.g., *Nasutitermes*). Common name groupings, based in biology, are meaningful in considering how to control termites. Understanding the biology of any pest is fundamental to successful control and even more critical when dealing with a social insect that can destroy people’s homes and their contents. For example, soil termiticide treatments and baits can be highly effective in subterranean termite control because the product is applied to where this termite lives, whereas a soil termiticide application for drywood termites would fail because they live in wood and do not forage into the soil.

Several decades of research have supported two categories (preventative and remedial) and four methods of control for termites: (1) construction practices (i.e., no wood-to-ground contact, the use of termite-resistant building construction materials), (2) termiticides (formulated as a liquid, gas, dusts, or bait), (3) treated building materials, and (4) physical barriers. What we now consider generally accepted control methods were viewed as alternatives to good building construction practices almost a century ago [2] (p. 503).

In the last 25 years, numerous review articles on the science behind termite control have been published [3,4,5,6,7,8,9,10]. The goal of this paper is to highlight the evolution of termite control by reviewing methods considered alternatives to the standard practice of the time, while noting the coevolution of regulatory processes that consider human health, environmental health, and product efficacy in contrast to home remedies and products that are not required to meet performance standards for structural protection.

## 2. The Recognition of Termites as a Pest

In an 1876 report of termite as pests, Hagen [11] chronicled a series of subterranean termite infestations in Europe and North America. In Rochefort, France, he noted that “…The danger (of structural collapse) was increased, as each owner carefully denied having these fearful guests, for fear of depreciating the value of his house”. He proceeds to describe how termites destroyed “costly timbers stored in the navy yard for building of men-of-war” (i.e., war ships) and “naval archives”. Scientific commissions were sent to study the “new pest” and devise solutions without success, “as refuse and manure spread the obnoxious insects further”. Attempts at chemical control were unsuccessful. Hagen [11] reported that only nonchemical methods provided some relief—”…the only way to avoid termite damage was “(c)onstant attention and the destruction of the pipes” (i.e., mud tubes), and the use of “only metal and stone” for new building construction.

Little has changed. Some key points from Hagen’s [11] narrative are as follows:

- Inspection is key to termite control.
- Building construction is a major variable of infestation. Building with termite-resistant construction materials is an important part of termite management plans.
- Termites not only damage structures, but also goods and contents.
- People are sometimes reticent to acknowledge termite risk to structures for fear of depreciating property value.
- Humans spread termites.
- The inability to adequately control termites with termiticides alone (“remedies”).

The large number of “remedies” were likely found insufficient because there was not yet enough known about subterranean termites as social insects and the potential population size of a colony. Hagen [11] refers to researchers studying what is now *Reticulitermes lucifugus* (Rossi) for about 100 years, but not in the context of a structural pest.

Before the use of mark–recapture techniques to estimate colony size, little was known about subterranean termite population and territory sizes. Destructive sampling methods produced population estimates for *Reticulitermes* species of 3000 to >100,000 [12] (p. 159) to 24,445 [13]. Destructive sampling includes direct counts, trapping, and core sampling of termite infested soil, but because of the subterranean nature of these termites, the location of a “nest” and the extent of the gallery system was largely unknown, making accurate estimates difficult.

Mark–recapture is considered a nondestructive sampling method that could provide an estimate of the foraging population. Pioneering studies in termite mark–recapture revealed that colonies of *Reticulitermes flavipes* (Kollar) and *Coptotermes formosanus* Shiraki can exceed estimates of 5 million [14] and 6.8 million individuals [15], respectively, covering territories as large as 2361 m^2^ [14], and 3571 m^2^ [15], respectively. The accuracy and validity of colony size estimates are not without controversy. Forschler and Townsend [16] found that termites extracted from logs were within the range of mark–release–recapture estimates, while Thorne et al. [17] and Evans et al. [18] did not. However, work in estimating colony size undoubtably enlightened our understanding of the potential for large termite pest populations. Pioneering researchers in Hagen’s [11] time did not have benefit of these data which likely led to frustrating failures in the pursuit of “remedies”. 

Hagen [11] hoped that “(w)hite ants (would) retreat step by step” with urbanization. We know this to be false. Early records indicated an increase in requests for termite control information over time. In 1916, the United States Department of Agriculture reported 37 requests for information on termites and how to control them. In 1918, there were 39 requests, and in 1919, there were 42 requests [19] (p. 95). Similar trends in increasing termite damage were reported in New York, where there was one infestation reported in 1932 with numbers increasing to greater than 12 in 1933. In 1934, substantially more infestations were reported, although no numbers were given [20]. In 1933, Jennings reported that losses due to termites were ~USD 29.3 [21].

Currently, there are 28 termite species that have been described as invasive, and these termites are spreading, partly due to global trade [22]. Evans et al. [22] noted that invasive termite species are associated with wood and that preventing introductions such as those used to contain the invasive Asian longhorned beetle, *Anoplophora glabripennis*, might serve as a model to stem the spread of invasive termites. However, the difference between these two pests is that the Asian longhorned beetle infests living trees, which is within the mission of several U.S. federal agencies, while most termites do not. Thus, no U.S. federal agency is tasked with surveillance for invasive termites, which is a critical step in pest prevention and making effective control measures all the more important.

## 3. Soil Termiticides Were Considered Alternatives to Good Building Construction in the First Half of the 20th Century

In 1927, a concerted effort to organize researchers working on termite control in the U.S. led to the formation of the Termite Investigation Committee. The formation of the Committee was in response to the “ravages of termites which (were) becoming increasingly destructive…” [23]. The efforts of the Committee and the pest control industry are summarized in the classic tome *Termites and Termite Control*, first published in 1934 [24] with a second edition in 1946 [25].

Soil termiticide treatments are the most commonly used method of subterranean termite control today, but in the first half of the 20th century, were considered an alternative to sound building construction. “…(T)he repair of faulty construction details, accompanied by a general clean-up of debris and scrapwood under the structure” and elimination of wood-to-ground contact was considered the primary method of termite control. Soil treatments were viewed “…as a promising ‘last resort’…where the costs of reconstruction do not seem justified” [2] (p. 503). Investigators observed that “soil poisoning” was mainly to prevent termite penetration of the treated soil, but that it would be “quite hopeless to expect…to kill the entire colony system” [2] (p. 504). They also observed that the thickness of the treated soil layer appeared to be more important than the concentration of termiticide; thus, soil treatments had to be thick enough so that the barrier would not be broken by “ordinary disturbances” [2] (p. 504). These principles still hold true today.

Compounds tested by the Committee for use as soil termiticides included Borax and magnesium fluosilicate at 5% and 10% at a rate of 1 gallon/10 square feet. These rates were found to be effective in preventing termite infestation. Randall and Doody [2] (p. 512) also recorded results from pest control companies that experimented with ammonium fluoride, sodium fluoride, sodium fluosilicate, and a kerosene solution of sodium arsenite. Proprietary products included: Antimite, a dry mixture of sodium fluoride, dinitrophenol, and sodium arsenate; Fluorex V and Fluorex S: different formulations of sodium fluosilicate and magnesium fluosilicate, respectively; and Terminix: a liquid mixture of orthodichlorobenezene, “toxic solvents, and toxic salts” applied to exposed wood and soil. The E. L. Bruce Company that produced Terminix also issued a five-year guarantee with their treatment and inspection service that also required all wood to be completely isolated from ground contact. It does not appear that any of these compounds had been tested by the Committee. None of these chemicals are currently registered and would be regarded harmful to the environment and human health. It is interesting to note that the rate of 1 gallon/10 square feet (for pretreatment soil surface treatment, termed a horizontal treatment) and the five-year guarantee are still used by the structural pest management industry today.

The Committee concluded that ground treatments should not be depended upon as a fundamental method of termite control [2] and were not considered a permanent remedy [26]. Nevertheless, soil termiticides flourished (Table 1).

Chlordane’s long residual [37], volatility [38], and cost-effectiveness were attractive characteristics in a soil termiticide, yet those attributes contributed to its demise and consideration as an environmental and human hazard. Soil treated 24 years earlier with a 1% solution of chlordane caused 88 to 94% mortality in Formosan subterranean termites after 5 days of constant exposure in bioassay [37]. The manufacturer withdrew chlordane in 1987 based on EPA’s risk assessment for human and environmental health [39].

Soil termiticides are no longer considered an alternative method of control, but we have witnessed a change in soil termiticide active ingredients. Potter [40,41] provides excellent overviews of historically used and currently registered soil termiticides. After the aforementioned cyclodienes (chlordane, aldrin, lindane, heptachlor; Insecticide Resistance Action Committee (IRAC) #2A), soil termiticides active ingredients came from the following insecticide classes: organophosphate (chlorpyrifos, isofenphos; IRAC#1B), pyrethroid (bifenthrin, cypermethrin, permethrin, fenvalerate; IRAC#3A), neonicotinoid (imidacloprid; IRAC#4A), phenylpyrazole (fipronil; IRAC#2B), and the newest liquid termiticide class, anthranilic diamide (chlorantraniliprole; IRAC#28). Products that combine active ingredients from different insecticide classes are also registered for use against subterranean termites.

## 4. Soil Termiticides: “Zones” as Alternatives to “Barrier”

In the 1990s, during industry training programs, we taught that there were two times a structure could be treated for termites: (1) at the preconstruction or new-construction phase or (2) post-construction, and that a good termiticide would be (1) toxic or repellent; (2) applied in a continuous barrier; and (3) last at least 5 years. These collective characteristics were built on decades of guidance that focused on maintaining a barrier to termites foraging into a structure. How did we transition from soil termiticide barriers to termiticides applied to “zones” for registration of termiticides that may provide less than 5 years residual activity?

### 4.1. Termiticide Repellency

Generally, repellency is defined as the quality or capacity of repelling [42], to drive back, or cause aversion [43]. Repellency with soil termiticides is a function of concentration. The pest management industry currently categorizes soil termiticides as “repellent” and “nonrepellent” based on whether subterranean termites penetrate soil treated at the termiticide label rate in laboratory tests. The value of repellency in an effective termiticide has been extolled as long as we have tested pesticides against termites [2,44]. Currently registered “repellent” termiticides contain pyrethroids (e.g., permethrin, bifenthrin) and their label directions instruct that the product be applied as an “…unbroken vertical and/or horizontal chemical barrier”. Interestingly, this language is the same that is used for products that contained chlordane [45]. We now know that chlordane likely acted as a fumigant termiticide in addition to its slower, concentration-dependent soil-bound toxicity. In theory, a “nonrepellent” termiticide is applied at a concentration that termites do not readily detect, so termites enter the treated soil (the zone), acquire a toxic dose, and die sometime after making contact with the zone. Nonrepellent termiticides contain the active ingredients imidacloprid, fipronil, chlorfenapyr, and chlorantraniliprole. Nonrepellents are discussed in Section 4.3, Product Innovation.

### 4.2. The Loss of Chlordane as a Driver of Research

Chlordane was withdrawn from the U.S. market in 1987, leaving the industry with termiticide products that were either organophosphates or pyrethroids. The 1990s were an uncertain time for termite control. Massive numbers of failures caused many pest control companies to flee the termite market [46], but it also drove innovation by manufacturers and researchers. Post-chlordane, several “repellent” products competed in the termiticide market. By 1991, 65.1% of pest management professionals (PMPs) surveyed indicated that Dursban TC (chlorpyrifos, then owned by DowElanco, Indianapolis, IN, USA) was their principal termiticide, followed by Demon TC with 12% of PMPs using it as their principle termiticide (cypermethrin, then ICI Americas), Pryfon at 10% (isofenphos, Mobay/Bayer), Dragnet FT at 8.5% (permethrin, FMC Corporation, Philadelphia, PA, USA), Torpedo at 2.1% (permethrin, then Roussel Bio), and Tribute at 1.8% (fenvalerate, then ICI Americas) [47]. There was also an increase in anecdotal reports of more “secondary colonies” after the loss of chlordane, which suggested insufficient control and thus termite control was reportedly “at a standstill” [48], but research was not. 

Prior to the loss of chlordane, Su et al. [49] had begun to categorize the active ingredients based not just on mortality, but also behavior.

- Type I termiticides included fenvalerate, permethrin, resmethrin, pyrethrins. These toxicants were viewed as repellent because the treated area was sealed off with little termite mortality.
- Type II termiticides included diazinon, chlorpyrifos, chlordane, carbaryl. Theses toxicants were less repellent. Termites were killed or affected quickly. The treated area was sealed off and tunnels contained dead termites.
- Type III toxicant included Amdro^®^, which was termed a slow-acting stomach poison. Tunnels were not sealed, and mortality increased over time.

Su et al. [49] concluded that Type I and II insecticides were fast-acting and repellent enough to prevent penetration into the treated soil; thus, best used for preventative treatments, and the slower-acting type III insecticide would be best for remedial treatments, including termite bait applications.

Part of advancing termite control was also understanding why the pyrethroids and organophosphates did not perform as well as the organochlorines. Numerous laboratory tests examined the repellency of termiticides [49,50,51,52], the ability of termites to find their way through untreated gaps of soil when repellent termiticides were used at various concentrations [53,54], as well as how treatment thickness [55] or population density influenced the ability of termites to breach a treatment [56]. Another factor that may have contributed to failures is that repellent termiticides did not result in significant mortality of test populations [49,51], so if termites were able to find a gap in treatment, they could still infest a structure. In contrast, chlordane caused significant mortality in laboratory bioassays with substrates treated in the field [37,57].

Additional factors that contributed to termiticide failures but were not related to termite behavior included enhanced microbial degradation or alkaline hydrolysis of registered organophosphates (isophenphos and chlorpyrifos, respectively), and the difference in termiticide application rates between chlordane versus pyrethroids and organophosphates. Rotramel [46,58] noted that pyrethroids and chlorpyrifos products were registered exactly at the rate that prevented termite infestations in the United States Department of Agriculture Forest Service (USDA-FS) termiticide tests located in Gulfport, Mississippi, unlike chlordane, which was registered at 1% [57]. Johnston et al. [57] reported that chlordane at 1/8% (0.125%) was 100% effective at preventing termites from infesting ground boards for 21 years.

The push to find effective yet more environmentally friendly termiticides fueled termite research in the 1990s through the early 2000s. This burst of research also contributed to improving efficacy testing. While screening new compounds in containers with small numbers of termites may provide preliminary data on efficacy, results from these types of tests should not be extrapolated to support claims of structural protection. Forschler [59] emphasized the need for a series of bioassays that incorporated termite behavior to provide more realistic results. Examples of studies performed to improve efficacy can be found in Table 2. 

Foraging behavior, tunneling, and factors affecting gallery formation became important as researchers attempted to understand the parameters affecting termite movement. Technological advances have allowed for the detailed examination of how termites excavate galleries. Excavation of galleries in soil is related directly to termiticide exposure and mortality. In one of the most detailed analyses of termite behavior yet, Whitman and Forschler [68] analyzed over 400 h of video in five behavior categories: mating, ecdysis, “tail-chasing”, feeding, and excavation. They observed that *R. flavipes* used their mouthparts only for manipulation of gallery substrate into a pellet (i.e., “pill” formation) as a first step in gallery formation, in contrast to Ebeling and Pence’s [69] earlier observations that reported *R. hesperus* Banks workers pushed their heads through moist sand, only carrying “smaller sand particles” in their buccal cavity and mixing these particles with a “gluey substance” before applying it to the tunnel. Whitman and Forschler [68] posited that pill formation, movement, and deposition would expose excavating workers to an oral dose of toxicant, leading to mortality. Forschler [59] demonstrated that oral exposure to soil termiticides led to more mortality than dermal exposure in most termiticides. Oral exposure would be less likely with “repellent” termiticides compared with “nonrepellent termiticides, because excavating termites would avoid the treated area.

In addition to Whitman and Forschler’s detailed study on gallery formation [68], examples of studies on termite foraging behavior included those that compared mark–recapture techniques [16,17], foraging populations of species other than those found in the U.S. [70], differences in foraging populations and worker physiology in urban and undisturbed habitats [71], distribution and habitats of the Formosan subterranean termite that involved pest management professionals in surveys [72], and finding a resource in relation to guidelines [73,74]. Numerous studies emerged on termite search patterns and tunneling behavior [75,76,77,78,79,80,81,82,83,84,85], including the impact of moisture levels on tunneling [86,87]. 

### 4.3. Product Innovation

In 1995, two innovative products were launched that became alternatives to repellent soil termiticides: Premise^®^ (a.i., imidacloprid, Bayer Environmental Science, Cary, NC, USA) was the first soil termiticide to be marketed as a “nonrepellent”. The second innovation was the commercial termite bait, Sentricon^®^ (Corteva, Wilmington, DE, USA, then DowElanco) (see Section 5. Baits, Wood Treatments as Alternatives to Soil Termiticides). In 2000, the termite control industry also welcomed Termidor^®^ (a.i., fipronil, now BASF Corporation, Research Triangle Park, NC, USA, then Aventis), marketed as a nonrepellent termiticide. By 2002, 48% of PMPs who responded to an industry survey indicated that they were using nonrepellent liquid termiticides versus 29% who were using repellent liquid termiticides. In addition, 68% of respondents said that their termite treatment revenues had increased while “only” 24% said that their termite retreatments had increased from the previous year [88] (p. S19). 

Nonrepellent soil termiticides are now an industry standard. In 2019, the average callback rate was 1.8% [89] (p. 6). Nonrepellent soil termiticides are sold under various brands or post-patent trade names and contain the active ingredients imidacloprid, fipronil, chlorfenapyr, and chlorantraniliprole. There is evidence of toxicant transfer with nonrepellent termiticides and resulting effects on behavior [90,91,92,93], but the degree of importance to termite suppression in field populations is equivocal. Nonrepellent termiticides can suppress termite populations and protect structures [94] likely due to termites coming into direct contact with treatments, but they have not consistently demonstrated elimination of populations from an area [95,96,97].

Changes to the termiticide product label language reflect what we now know about the reality of applying soil termiticides to an active building construction site, changes in active ingredients, and subterranean termite behavior. For example, the term “barrier” implies that the treatment is impenetrable. While repellent termiticides can be impenetrable in laboratory studies, gaps of untreated soil can occur on an active construction site. In laboratory studies, gaps in repellent treatments allowed termites to reach food sources with low mortality compared with an inability of termites to find untreated gaps and reach food sources, plus high mortality was observed in nonrepellent termiticide treatments [54,98]. Thus, the term “barrier”, found in labels of repellent termiticides, has been changed to “treatment zone” on nonrepellent termiticide labels to reflect that termites enter the treated area and that there may be a degree of population suppression. Application rates have remained consistent for decades, with most termiticide labels requiring 1 gallon/10 square feet for horizontal areas, 4 gallons per 10 linear feet per foot of depth for vertical treatment areas, and 2 gallons per 10 linear feet for hollow block or other masonry wall units.

As product chemistries continue to evolve, so do pesticide regulations. For example, in the same year that Termidor™ was launched, chlorpyrifos was lost from the structural pest control industry. Chlorpyrifos was available to the industry for about 20 years after receiving a conditional registration as a termiticide in 1980 [99]. In 2000, the U.S. EPA announced that it was taking “the fastest action possible” to remove chlorpyrifos-containing products in response to the passage of the Food Quality Protection Act of 1996 [100]. The Food Quality Protection Act changed how cumulative risk due to pesticides was calculated so that estimates became more conservative. Part of the U.S. EPA’s intent was to “move to newer, safer alternatives”, to which termiticide manufacturers, researchers, and industry experts responded through continual process of innovation.

Product registration in the U.S. is highly regulated for pesticides claiming structural protection. The U.S. EPA has product performance standards intended to protect structures associated with the registration of soil termiticides (see Section 7. Product Performance Standards and Pesticide Labels Leave Little Room for Alternatives).

## 5. Baits and Wood Treatments as Alternatives to Termiticides

Baits, wood treatments, particle size barriers, marine-grade stainless-steel mesh, and polyethylene sheets with and without termiticides are being used as alternatives to, or in conjunction with, soil termiticides and they have been thoroughly reviewed [3,5,7,10,69,101,102,103,104,105,106,107,108,109,110,111,112]. What has not been discussed is how these products may differ in regulatory aspects. Baits and wood treatments are considered pesticides, and therefore, within the regulatory authority of the U.S. EPA (nonchemical methods are covered in Section 6. Physical Barriers as Alternatives to Termiticides). 

When structural protection claims are made, the U.S. EPA provides additional product testing and performance guidelines for soil termiticides and wood treatments that include sprays, pressure treatments, dips, and brush-on applications for subterranean, drywood, and dampwood termites (OPPTS 810.3600) [113] and baits (OPPTS 810.3800) [114] (see Section 7. Product Performance Standards and Pesticide Labels Leave Little Room for Alternatives). Some states, such as Florida, also have additional regulatory requirements, such as 5E-2.0311 FAC [115]. 

### 5.1. Termite Baits

Evans and Iabal [106] and Su [108] recently reviewed the development of baits. The U.S. EPA also includes a list of publications supporting the test guidelines (OPPTS 810.3800) [114]. It is important to note that some over-the-counter products intended for sale to nonprofessionals do not make claims of structural protection. Popular baits intended for professional use with claims of structural protection in the U.S. contain insect growth regulators that are incorporated into a cellulose matrix and installed in the soil for subterranean termites. For example, Sentricon^®^ (Corteva, Wilmington, DE, USA) and Trelona™ (BASF, Research Triangle Park, NC, USA) contain the active ingredients novaflumuron and novaluron, respectively. Pest management professionals often prefer U.S. EPA-registered baits over liquid soil termiticides because they can be installed based on simple linear footage (e.g., every 10 to 20 feet) and not by gallons required for a site that is calculated by totaling the amount needed to treat horizontal surfaces (square footage), and vertical areas (linear footage per foot of depth, void, and critical areas). 

Baits are effective in structures with complicated construction. In theory, baits eliminate or suppress termites from an area, thereby decreasing or eliminating risk to the structure. Collectively, both products have a significant body of research supporting the concept of termite colony elimination and suppression in structures with existing infestations [116,117,118,119,120,121]. Sentricon™, specifically, has been successfully used in high-profile and historic sites such as the Statue of Liberty [122], Cabildo Complex of French Quarter, New Orleans [123], El Morro and San Cristóbal at the San Juan National Historic Site [124], Fort Christiansvaern, Christiansted National Historic Site, St. Croix [125], Tzu-Su Temple, of San Shia, Taiwan [126], and Madame John’s Legacy House [127].

Baits are being used as a preventative method of control in new construction. Installation is usually carried out after the structure is constructed and during the final grading of the site, when the landscape is installed. While baits can be effective, as with all termite control products, there are limitations to efficacy. In the case of baits, some limitations seem to be related to regulatory requirements of when baits are installed during the new construction process. If construction takes months or a year to complete, and baits are not installed until the final grade, a home or structure may be infested before occupancy because there is no subterranean termite protection. There are anecdotal reports of this exact situation. A second limitation of baits is if the stations are removed inadvertently or because the owner no longer wishes to maintain the contract, which prompts the removal of the bait stations, leaving the property without termite protection. A third limitation is that bait stations placed uniformly may not intercept termites efficiently. Placement in areas of conducive conditions will increase the number of stations infested and shorten the amount of time for termites to discover stations [128]. Innovations in baits continue. Fluid baits have been tested [129,130], but are not yet commercialized.

Interestingly, early efforts at control included fluid baits, in addition to trapping, spraying infested timbers, and the injection of poison dusts for subterranean termite control. All were considered failures at the time [131] (early investigators also knew that fumigation and heat treatments were not effective for subterranean termite control because the treatments were not residual [131]). Early attempts at baiting seemed to be better suited for ants than termites. Anecdotal reports of “straw or chaff soaked in a solution of sugar and sodium arsenite” as a bait against the harvester termite in the tropics, and a bait of 28 g white arsenic or sodium arsenite mixed with 454 g of “treacle” (probably a sugar-based syrup) poured into woodwork was reportedly used to control termites in Australia [2] (p. 475). Randall and Doody [2], however, did not find that drywood, dampwood, or subterranean termites would feed on baits of 10% white arsenic and honey or 0.5% sodium arsenite in a dilute sugar solution. The termites appeared to avoid sugar-based baits. It would be decades before Sentricon^®^, the first commercially available termite bait, was launched in 1995. 

### 5.2. Wood Treatments

In 1898, Dr. Karl Henrich Wolman developed the Wolman salts as a wood preservative. In conjunction with the Antimite Company, Wolman also developed a product for termite control which used the Wolman salts. Records as far back as 1925 indicate that the Antimite Company was one of the first companies exclusively “engaged in helping the exterminator to solve the problems of termite control work” [132] (p. 14). Borates have a long history in structural protection, including their use in termite control, and have been reviewed by Williams [133,134] and Grace [102].

*Bora-Care^®^ as a case-study of regulatory contradictions*. Many liquid termiticides include wood treatments that supplement another termite control method. However, Bora-Care^®^ (Nisus Corporation, Rockford, TN) claim in their labeling that it is the only borate termiticide that passed the U.S. EPA’s requirements as a standalone treatment for new construction [135]. Other companies were able to legally cite Nisus’ data without conducting efficacy studies under the U.S. EPA’s Data Compensation Requirements [136]. U.S. EPA’s guidance for wood treatments suggests that a product should provide complete resistance to termites for 2 to 5 years with annual inspection (OPPTS 810.3600) [113]. A *suggested* standard leaves room for dialog between the Agency and manufacturer. Here, I also introduce a second state-level requirement as background on how regulatory interpretations can affect product registrations: The Florida Rule. In March 2003, the Florida Department of Agriculture and Consumer Services (FDACS) adopted the Termiticide Efficacy Rule, titled Performance Standards and Acceptable Test Conditions for Preventive Termite Treatments for New Construction (5E-2.0311 of Florida Administrative Code (FAC)) [115]. The Florida Rule requires registrants to provide data to FDACS demonstrating that their product protects 90% or greater of experimental units for at least 5 years for pesticides applied to wood. Both agencies accept field plot and building tests as part of the data packet for product review.

Sequence of events: On 6 March 2006, FDACS Scientific Evaluation Section issued its findings, allowing Bora-Care^®^ as a standalone new construction treatment in Florida [137]. Both field tests and building tests are required to meet Florida Rule performance standards (5E-2.0311 (2)(c)(1) FAC) [115]. A little over a year later, in June 2007, the U.S. EPA issued their Product Performance/Efficacy Review, stating that “(i)n their whole, the data provided are adequate to support a structural pretreatment claim against subterranean termites (*Reticulitermes* spp. only)” [138].

The FDACS decision was based on two studies: field joist tests and building tests. The U.S. EPA data package included several additional studies, including a building test that appeared to be a common dataset in both packets (MRID 46753005) [137,138]. Building tests were performed in cooperation with a large pest control company. The dataset in both packets were collected from 32 properties. The description of results is similar; however, the U.S. EPA *rejected* this study, stating that just one of the 32 structures “could be used to validate the effectiveness of Bora-Care^®^ in preventing infestation of a treated structure” and the product failed in that single structure, while FDACS found the study to meet the Florida performance criteria.

The weakness of 5E-2.0311 FAC is apparent when analyzing how FDACS concluded that Bora-Care^®^ passed the performance criteria of the Florida Rule. The FDACS report stated that the registrant randomly selected homes from a pool of 98 [137]. The properties were treated in 2000, which was before the adoption of the Florida Rule in 2003. According to the Florida Rule, there is no requirement for termite activity in building tests of wood treatments installed prior to the adoption of the Rule (5E-2.0311 (2)(c)(9)), although tests installed after the adoption of the Rule are required to demonstrate termite activity within 10 feet of the structure in a minimum of 10 structures 5E-2.0311 (2)(c)(10). Thus, if tests were started before the adoption of the Florida Rule, it would be acceptable to submit data from a site that did not have any termite activity. In contrast, U.S. EPA performance guidelines require termite activity at test sites [113]. The requirement for termite activity is the crucial difference in how the same dataset was approved by FDACS and rejected by the U.S. EPA.

In 2004, the registrant complied with FDACS’ request to voluntarily provide a measure of termite activity. Two wooden stakes were installed, one each at opposite ends of the structures. In the final report, one structure had termite activity within 10 feet of the structure in one wooden stake. Termite activity was identified in other wooden debris on four additional properties, but not within 10 feet of the structure. The termites in the single wooden stake were identified as *R. flavipes*. Additionally, two structures were disqualified because they had been treated with a soil termiticide “in response to termite activity”. The registrant could not determine “whether the infestation was the result of misapplication of Bora-Care or due to product failure”, and two structures were replaced with two others from the original pool [137]. One other structure had termite activity and was deemed a failure. The wooden stakes at the failed structure did not detect termite activity. Based on the Florida performance criteria for wood treatment tests installed before the adoption of the Florida Rule, FDACS calculated a 4% failure rate (1 failure out of 27 structures) within 5 years, thus passing the Florida performance criteria.

Presence of termites should be a condition of every product test. An artificial construct of whether termite activity should be required in tests carried out before or after the adoption of the Florida Rule influenced how the data were interpreted under 5E-2.0311 FAC, which affected how failure rates were calculated, thereby impacting the registration process. If the same Bora-Care^®^ product was tested after the adoption of the 2003 Florida Rule, the registrant would have been required to demonstrate termite activity within 10 feet of a structure in a minimum of 10 of 25 structures, according to 5E-2.0311 (2)(c)(10) FAC. Submitting a single structure with demonstrated termite activity would have disqualified the submission packet to FDACS. In the final report, FDACS also had broadly interpreted termite activity on the properties to include debris farther than 10 feet from the structure, increasing the sample size to five properties with activity, but it would still not have met the Florida Rule in term of numbers of properties requiring termites. If the failure rate was calculated based on properties with demonstrated termite activity, this product would still have failed the Florida Rule performance criteria of 90% or greater of test structures being termite-free for 5 years after treatment. The product failure rate was 16.7% (one failure of six properties with termite activity), and the success rate was 83.3%.

The Florida Rule does not require testing against different subterranean termite species. FDACS conclusions were based on *Reticulitermes* spp., but Bora-Care^®^ is used on homes where *Coptotermes* spp., and occasionally *Nasutitermes corniger* also, are found. The Florida Rule has not been modified since 2003 and it does not contain provisions for periodic product reviews. In 2007, the U.S. EPA requested that the registrant add “This product will not provide structural protection against/from the Formosan subterranean termite, *Coptotermes formosanus*” [139]. However, this request was followed by the conditional acceptance of a label amendment which included a request for additional data supporting the claims for preventative treatments in protecting against the Formosan subterranean termite [140]. Nisus provided unpublished data to the U.S. EPA’s satisfaction. The U.S. EPA archives did not contain information on the data submitted to support claims for protection against the Formosan subterranean termite. The Bora-Care^®^ label does not include *Nasutititermes*. Peters and Fitzgerald [141] stated that borates are not a “viable management option” based on Gay et al. (1958) [142], but a current borate formulation tested against *N. corniger* could produce different results. Peters and Fitzgerald [141] also suggest that untreated wood may serve to decrease the efficacy of borates by what is most easily described as a “dilution effect” in *Coptotermes* spp. Peters and Fitzgerald used a sodium polyborate complex that was vacuum-impregnated into pine, which is different than Bora-Care^®^’s active ingredient disodium octaborate tetrahydrate that is mixed in a proprietary blend of glycols. These differences prompt the need for more research, particularly with *Nasutitermes* spp., and begs the question of what happens to homes treated with borates if *Coptotermes* or *Nasutitermes* termites move into their neighborhood post-construction. 

The Florida Rule is dated and a revision has been suggested [143]. It is difficult to know how Bora-Care^®^ has fared over time as a standalone treatment. There are no publicly available follow-up reports on structures treated with Bora-Care^®^ only, and many companies offer homeowners a perimeter treatment with a soil termiticide at the one-year renewal mark; hence, a second treatment would confound these data. In 2021, promotional material “For Building Pros” indicated that more than 2,000,000 homes had been treated [144]. The product offers a 30-year limited warranty for new-construction single family homes up to USD 2500 as reimbursement for damages and free product for retreatment if a company meets certain conditions [145]. 

## 6. Physical Barriers as Alternatives to Termiticides

Particle size barriers, stainless-steel mesh, and polyethylene barriers are nonchemical methods that may be considered “devices” by the U.S. EPA and are subject to regulation but not pesticide registration requirements [146,147]. Each of the methods listed is considered a more environmentally friendly alternative to soil termiticides, using significantly less to no pesticides. 

While there is research to support the efficacy of nonchemical methods, there is not a regulatory network equivalent to the collaboration between the U.S. EPA, USDA Forest Service, and state lead agencies that ensures structural protection for consumers. U.S. EPA-registered baits and wood treatment products are required to have pesticide labels with use directions that can be enforced by federal and state regulators. Nonchemical methods may have detailed installation directions, but there are no regulatory agencies tasked with ensuring their proper installation or use. U.S. building codes reference termite protection, including nonchemical methods, but building inspectors are not tasked with ensuring that termite protection is installed or used correctly. So, while imperfect, the regulatory structure that baits and wood treatments must work within seems to be absent with nonchemical methods of termite control. 

Physical barriers, such as particle size barriers and marine-grade stainless-steel mesh, are commercially available as nonchemical termite control devices. Nonchemical methods are installed as a part of the building construction process. Products can be part of a system that includes physical barriers around the perimeter of the building, fittings around pipes, and special sealants. Proper installation is critically important to the performance of these devices. The U.S. EPA considers any device used for pest control to be subject to regulation under FIFRA.

The Agency defines a device as “any instrument or contrivance (other than a firearm) that is intended for trapping, destroying, repelling, or mitigating any pest or any other form of plant or animal life (other than man and other than bacteria, virus, or other microorganism on or in living man or other living animals); but not including equipment used for the application of pesticides when sold separately there from.” [146]. Pest control devices do not undergo the U.S. EPA pesticide registration process, but an EPA Establishment Number is required, depending on claims being made. The U.S. EPA does not require efficacy or safety data to receive an establishment number. The seller is responsible for product performance. The U.S. EPA also notes that states may have additional requirements, and sellers of the device should consult with state lead agencies to confirm that their device can be legally sold in the state [147].

Regulatory oversight can provide some level of consumer protection. The presence of an EPA Establishment Number is one way to separate products that are subject to regulatory oversight from those that are not. In the case of steel mesh, the International Code Council (ICC) has Acceptance Criteria for Termite Physical Barrier Systems (AC380). However, there is still a lack of regulatory oversight at the point of installation for all nonchemical methods of termite control.

### 6.1. Particle Size Barriers

The concept of particle size barriers as a termite control method is commonly attributed to Ebeling and Pence [69], who observed that *R. hesperus*, the western subterranean termite, could not penetrate sand particles that were ~1.2 to 1.7 mm in diameter in the laboratory. Similar to chemical barriers of that time, the principle behind particle size barriers was to create a continuous barrier to exclude termites from entering a structure. Ideally, the particle sizes had to be large enough so that termite mandibles could not grasp it, yet packed tightly enough so the interstitial spaces were too small for termites to squeeze through. Tamashiro et al. [107] noted that in order to be practical, a mixed range of particle sizes had to be effective in preventing termite infestations, and the material also had to be heavy enough so that termite workers would not be able to move the particles should they be able to grasp some of the particles with their mandibles. Finally, the material had to be resilient enough to not crush under the weight of the structure [107]. Through a series of laboratory and field tests that lasted 4 years, Tamashiro et al. [107] demonstrated that basaltic rock particles >1.7 and <2.8 mm were effective in excluding Formosan subterranean termites. Tamashiro et al. [107] also noted that granite was successfully substituted in Australia (French, 1989, personal communication in Tamashiro et al. [107]) and was developed into Granitgard™ [148].

Yates et al. [103] reviewed the research and development behind the Basaltic Termite Barrier that was first commercialized by Ameron HC&D. The Basaltic Termite Barrier patent expired (U.S. Patent 5,094,045), but similar particle size barriers continued to be commercially available, including Granitgard™ [148] and TERM^®^ Particle Barrier that is sold as part of the TERM^®^ Barrier System (EPA Establishment No. 89537-TX-1, Polyguard, Ennis, TX, USA) [149]. TERM^®^ Particle Barrier was part of the submission for EPA Establishment Number No. 89537-TX-1; it is not a component listed in the ICC-ES Evaluation Report ERS-3632 [150]. A national list of active EPA registered devices can be found online [151].

Granitgard™ is sold as part of the termite management system in Australia. An additional product includes Blockaid-Termi, a sealant that contains bifenthrin. Granitgard™ has been commercially available since 1992 and has been installed in >200,000 homes. It is nationally certified, meets the building code standards of Australia, and offers a warranty for up to 50 years with conditions that include an annual inspection at the owner’s expense [152]. The termite species in Australia are more varied than in the U.S., thus the particle size range varies slightly from the particle size range identified by Tamashiro et al. [107]. Methods of termite control in Australia, including termite barriers, were reviewed by Ahmed et al. [109]. Ahmed et al. [109] cautioned that termites can tube over barriers, so regular inspections remain an essential component of termite management. Numerous studies have confirmed the effectiveness of particle size barriers against termites of economic importance [110,153,154,155,156], but adoption remains low, partially due to cost and product availability. Other limitations include contamination of the product with particles of other sizes, uneven soil compaction under a slab, and failure to maintain a 4-inch layer of Basaltic Termite Barrier under the slab which compromises performance [103].

### 6.2. Marine-Grade Stainless-Steel Mesh

Termimesh was invented in Perth, Australia in 1989. Termimesh is part of a system that includes adhesives for various surfaces (Termiparge, Termibond), prefabricated collars for pipe penetrations (Termistop), and signage (Termitape) to indicate the presence of Termistop to other trades working on the construction site. The concept of leaving signage to remind other trades of termite protection on each slab penetration is novel. Termimesh meets building code requirements of the International Code Council (AC380). It carries the U.S. EPA Establishment Number 083929-TX-001 and carries CodeMark Australia certificate number CM30012 Rev 3 [157]. CodeMark Australia is a third-party auditor that facilitates compliance with the Building Code of Australia [158].

The product has evolved. Lenz and Runko [111] reported that the product was initially tested with 304 stainless steel. The company then began production with a higher grade 316 stainless steel. Currently, Termimesh’s stainless steel (TMA725) is proprietary, containing twice the molybdenum of 316 stainless steel [159], exceeding the requirements of 316 stainless steel. Molybdenum increases resistance to corrosion.

The aperture size of the mesh is 0.66 by 0.45 mm or 0.45 by 0.45 mm for areas with *Heterotermes vagus* (Hill). The diameter of the wire is 0.18 mm. In early studies, Lenz and Runko [111] demonstrated that wood was protected from *Coptotermes acinaciformis* (Froggatt), *Mastotermes darwinensis* (Froggatt), and *Schedorhinotermes breinli* (Hill) in Australia after 3 years of field testing. Grace et al. [101] found the product effective after 1 year of field testing against *C. formosanus.* As with other methods of termite control, attention to detail in application or installation is key. Grace et al. [101] noted that Formosan subterranean termites gained access to one test unit by breaching the bonding cement used to hold a thick fold of mesh, which is not the normal use of these components during building construction. Kard [104,105] reported 100% efficacy against subterranean termites after 5 years of field testing at the U.S. Forest Service sites in Arizona, Florida, Mississippi, and South Carolina. The U.S. Forest Service sites had 90 to 100% attack on wood at untreated control plots. Based on the most current U.S. Forest Service report [160], it appears that none of the soil termiticides except Biflex TC (i.e., bifenthrin) and Termidor^®^ SC (a.i., fipronil) can claim 100% efficacy at the common use rate under the EPA guidelines of 5 years or more at all U.S. Forest Service testing sites. Termimesh warranties vary by country, and in the U.S., by state.

### 6.3. Other Barrier Systems

Polyethylene barriers impregnated with insecticides have been effective in termite prevention. Su et al. [112] found that polyethylene impregnated with lambda-cyhalothrin was effective for >5.5 years after installation under concrete slabs against *C. formosanus* and *R. flavipes*. Baker et al. [161] found that this product, Impasse^®^, to be similarly effective against *Heterotermes aureus* (Snyder) and *Gnathamitermes perplexus* (Banks in Banks & Snyder, 1920) after 6 years of field testing. Wagner [162] reported that none of the impregnated barriers failed in the U.S. Forest Service trials. Kordon Blanket was installed in 1999 and contained deltamethrin. Termi-Film was installed in 1998 and contained permethrin. Impasse^®^ was installed in 1999 and contained lambda-cyhalothrin. The final product, A + Protect^®^ was installed in 2001, but it does not appear that the product or company exists. The active ingredient for A + Protect could not be found. Impasse ^®^ has also been discontinued. Kordon^®^ is now the Kordon Termite System AU [163].

Ewart [164] credits Termi-Film^®^ (then Cecil, now sold by Adkalis [165]) as the first plastic sheet product impregnated with a termiticide, permethrin. He also lists polyethylene product HomeGuard^®^, impregnated with bifenthrin, and Trithor^®^, impregnated with deltamethrin [166]. 

It is difficult to assess the effectiveness of nonchemical, plastic sheet barrier systems. Some systems, such as Term^®^ Barrier System (Polyguard Products, Inc.), provide ICC-ES Evaluation Reports [150] as evidence of being compliant with building codes in the area of termite protection. However, there is no equivalent dataset on product performance to be compared with other termite control methods. Nonchemical methods of termite control should meet the same performance standards required of pesticides, but in the U.S., these products fall into a regulatory gap, leaving consumers at risk. While some companies seek independent research on the efficacy of their products, the lack of regulatory oversight leaves the potential for companies to make claims of structural protection without meeting performance standards that pesticides must meet.

## 7. Product Performance Standards and Pesticide Labels Leave Little Room for Alternatives

Pesticide registrations are one responsibility of the U.S. EPA. The product registration process is lengthy and expensive. In addition to the U.S. EPA’s evaluation process briefly described below, termiticides also must meet the labeling requirements of Pesticide Registration Notice (PRN) 96-7 [167] and Product Performance Test Guidelines published subsequently by the Office of Prevention, Pesticides and Toxic Substances: OPPTS 810.3600 Structural Treatments [113] or OPPTS 810.3800 Methods for Efficacy Testing of Termite Baits [114].

For clarity, the U.S. EPA distinguishes between the “label” and “labeling”. “Label” and “labeling” are used in the context of these definitions: The “label” is defined as “the written, printed, or graphic matter on, or attached to, the pesticide or device or any of its containers or wrappers”. “Labeling” is defined as “all labels and all other written, printed, or graphic matter” [168].

Pesticide labels are a critical component of the product registration process. They provide information on how to use products effectively while protecting human and environmental health. All pesticide labels, including termiticides, carry the statement “It is a violation of Federal law to use this product in a manner inconsistent with its labeling”. Thus, in the U.S., there is a common saying, “the label is the law”.

Some of the information manufacturers must provide as part of the registration submission packet for any pesticide includes [169]:

- “Data on potential risks to human health and the environment….
- Proof that the product manufacturing process is reliable.
- Labeling, including directions for use, contents, and appropriate warnings”.
- Some of EPA’s evaluation process include:
- “Human health risks (including sensitive groups such as children and immune-suppressed individuals), by reviewing data on:
  ○ Aggregate risks through food, water, and residential uses
  ○ Cumulative risks from different pesticides with the same effects
  ○ Occupational risks to those applying the product during their work
- Environmental risks by reviewing data on:
  ○ Potential for ground water contamination
  ○ Risks to endangered and threatened species
  ○ Potential for endocrine-disruption effects…”

In 1996, the U.S. EPA issued PRN 96-7 specifically on termiticide labeling [167]. It was created “(b)ecause of the highly specialized nature of termiticides (and) a number of labeling issues (had) evolved over the years regarding: (1) limitations on distribution, sale or use; (2) precautionary statements; (3) environmental hazards statements; (4) storage and disposal statements; (5) use directions; (6) the minimum product performance of termiticide treatments; and (7) application at less than labeled rates”.

Labels for currently registered soil termiticides are intended for commercial applicators, even though they are accessible to the public, because they are labeled as “general use products”. Commercial applicators are subject to training requirements that non-pest management professionals are not. The U.S. EPA has recommended that the following statement be added to termiticide labels: “For use by individuals/firms licensed or registered by the state to apply termiticide products. States may have more restrictive requirements regarding qualifications of persons using this product. Consult the structural pest control regulatory agency of your state prior to use of this product” [167].

Striking a balance between product efficacy and environmental considerations has significantly impacted product registrations for termite control. Section IV of PRN 96-7 addresses efficacy, stating that the current Agency’s policy is that soil termiticides “should demonstrate efficacy for at least five years against termites”. As justification, PRN 96-7 states that “(t)he most recent data from the USDA Gulfport Mississippi Laboratory indicate that most currently registered products are effective for three to five or more years” [167]. It also states that the Agency will not grant registration to a soil termiticide product that requires an annual retreatment unless “*the pesticide is either significantly less toxic than currently registered pesticides or the benefits from the use of the pesticide are much greater than currently registered alternatives*”. These statements allow the Agency leeway in approving products that submit less than 5 years of data.

The Product Performance Test Guidelines OPPTS 810.3600 [113] specifies methods and suggested performance standards for soil termiticides and preventative wood treatments. The Guidelines are to ensure the efficacy of products in protecting structures against termite damage. The suggested performance standards for soil termiticides are that the data should demonstrate “complete resistance to termite attack for a period of 5 years, based upon annual reinspection”. The suggested performance standards for wood treatments states that products should demonstrate “least 2 years but less than 5 years” of “complete resistance to termite attack, the product may be registered contingent upon a restriction which specifies annual reinspection”. Performance standards for soil termiticides and wood treatments state that the “tests should be in geographical areas which provide year-around pest pressure (Usually in the southern U.S.)”. 

Practically speaking, termiticide registrations are highly dependent on the judgment of the U.S. EPA data packet evaluator, who must consider U.S. EPA’s general pesticide registration requirements [169], PRN 96-7 [167], OPPTS 810.3600 [113], or OPPTS 810.3800 [114]. Products are considered on a case-by-case basis and should not have to be applied annually to confer structural protection. While the process is imperfect, there is an evaluation process and suggested performance standards that “kills only” products (i.e., products that do not claim structural protection) and home remedies are not required to meet.

The availability of professional products on the Internet and free videos demonstrating how to use these products, combined with the expense of professional termite treatments, can lead property owners to think that they can carry out termite treatments by themselves. The regulatory language limiting the sale of professional termite control products to licensed individuals or companies is not a deterrent to unlicensed individuals who seek to purchase them. It is important to recognize that if products that meet this performance standard are applied according to the label directions, structural protection can be expected regardless of whether the applicator was credentialed or not.

## 8. Building Codes Leave Little Room for Termite Control Alternatives

Protecting structures from termites has been in building codes for almost 100 years. In 1923, Burlington, Iowa, was the first to include termite prevention as part of their building code [26]. In 1927, the Pacific Code Building Officials followed suit by adopting the Bureau of Entomology and Plant Quarantine recommendations for termite damage prevention [26]. In 1928, the city of Honolulu, Hawaii, adopted similar provisions [26]. Building codes developed concurrently with soil termiticide treatments.

Termites, particularly subterranean termites, are recognized by building codes as a threat to structures. Building codes contain sections of termite protection language. For example, the 2018 International Building Code contains Section 2304.12, Protection Against Decay and Termites under General Construction Requirements [170]. States with high levels of termite pressure, such as Florida, contain specific sections (R318, Protection Against Termites) on termite protection in their building codes that leave little room for unproven alternative termite control methods [171]:

“Termite protection shall be provided by registered termiticides, including soil applied pesticides, baiting systems, and pesticides applied to wood, or other approved methods of termite protection labeled for use as a preventative treatment to new construction. See Section 202, “Registered termiticide”. Upon completion of the application of the termite protective treatment, a Certificate of Compliance shall be issued to the building department by the licensed pest control company that contains the following statement: “The building has received a complete treatment for the prevention of subterranean termites. Treatment is in accordance with rules and laws established by the Florida Department of Agriculture and Consumer Services”.

[171]

In addition to specifying that registered termiticides should be used, Florida Building Code Section R318 also contains specific requirements of 6-inch inspection space between the siding and grade, requires gutters and downspouts to discharge at least one foot from the foundation, specifies that soil termiticide treatments must be carried out after compaction and that if an area is disturbed, the area must be retreated, and that a vapor barrier must be installed after a soil termiticide treatment [171]. The Florida Building Code does not specify who must contact the pest control company to do the retreatment if the soil is disturbed; thus, the potential for miscommunication exists. The Florida Building Code also references sections of the Florida Administrative Code (FAC) that require the pest control company to provide a warranty to homeowners for up to 5 years after a treatment for new construction (5E-14.105(3)(a,b)) [172]. Termite treatments are heavily regulated.

Some nonchemical physical barriers have obtained ICC-ES^®^ or CodeMark™ product certifications to demonstrate that their products meet building code requirements (see Section 6 Physical Barriers as Alternatives to Soil Termiticides).

## 9. Registered Products without Claims of Structural Protection, Minimum Risk Pesticides (FIFRA 25(b)), and Home Remedies as Alternatives to Professional Products

There are three other categories where termite control products can be found: U.S. EPA registered products that do not claim structural protection, also known as “kills only”, minimum-risk pesticides designated as “exempt” from registration under FIFRA 25(b), and “home remedies”. Products in these categories are used by the public as alternatives to professional products and services. These products entirely shift the liability to the user.

### 9.1. Registered Products without Claims of Structural Protection

“Kills only” termiticides have been defined as “a pesticide product that kills termites when applied to active infestations but does not produce significant residual activity that will prevent subsequent reinfestation” [173]. These pesticides are also registered by the U.S. EPA but are not subject to the performance standards required by termiticides that make structural protection claims. They may contain the same active ingredients as professional products which may lead non-pest management professionals to think that they can achieve structural protection. However, the average person may miss the important warnings in small print stating that retail products are “not intended to provide structural pest control” when compared to the large marketing print claiming the product is “(e)asy to use” and after application one could simply “walk away” [174]. Non-pest management professionals may not understand that killing a few foraging termites is not enough to protect their property.

Marketing language that claims the product “kills foraging termites” while simultaneously stating in small print that one should contact a professional for active infestations contributes to consumer confusion [175]. Other warnings in small print include that “(t)he Buyer assumes responsibility for lack of performance or safety if not used according to the directions”, the product “can only be used to supplement a federally registered conventional product that is registered as sole source for termite control”, and it “will not eliminate termite infestations or provide protection against future termite infestations” [174]. Warnings in small print such as these may contribute to distrust over claims of product efficacy. It would be interesting to explore whether claims that contribute to distrust are a factor in driving consumers to do-it-yourself solutions.

### 9.2. Minimum Risk Pesticides (FIFRA 25(b))

Some pesticides have been exempt from registration because they pose little risk to human and environmental health [176]. These are sometimes referred to as “25b’s”, which stands for the statute Federal Insecticide Fungicide Rodenticide Act (FIFRA) 25(b). It is possible that exempt products can kill termites, but none contain active ingredients that meet the performance standards for structural protection [176]. Examples of active ingredients on the minimum-risk pesticide product list include several essential oils, corn gluten meal, corn mint, dried blood, eugenol, putrescent whole egg solids, sodium lauryl sulfate, white pepper, and zinc.

### 9.3. Home Remedies

There are an unlimited number of home remedies on the Internet for termite control. Pinterest (https://www.pinterest.com/) (accessed on 24 November 2021) is a popular platform for the do-it-yourself (DIY) community. It runs on a visual discovery system based on pictures instead of text [177]. I entered “how to get rid of termites”, a search term that a non-pest professional might enter. The first board, 23 Home Remedies for Termites [178], included the following recommendations, each with explanations and commentary on why each “remedy” would result in termite control. I list the 23 recommendations here because the subsequent 8 boards (the virtual place where information (“pins”) is saved and organized) that I reviewed generally repeated a tedious litany of similar ineffective methods. Home remedies were to “remove stumps”, “ change your landscape”, “flood them out—a natural termite killer”, “orange oil”, “neem oil”, “clove oil”, “garlic oil—the ultimate natural termite killer”, “cardboard” (trap and remove), “diatomaceous Earth”, “boric acid”, “white vinegar—one of the easiest home remedies for termites”, “soapy water—easy homemade termite killer”, “aloe vera”, “petroleum jelly”, “canola oil”, “salt”, “parasitic nematodes”, “*Beauveria bassiana*”, “sunlight”, “heat treatment”, “cold treatment”, and “termite barrier—an easy preventative tip”.

Details for applying the clove oil remedy included placing 3 drops of clove oil and ½ cup of water in a spray bottle to apply on the termites. Similar guidance was given for the white vinegar remedy: place 2 tablespoons white vinegar, 1 teaspoon lemon juice, and ½ cup of water in a spray bottle [178].

- Directions for the cardboard remedy were to “(P)lace the damp cardboard box in strategic areas near the wooden structures in or around your home and the termites will be drawn to it. Once you notice termites in and on the box, destroy it”.
- Aloe vera: “Crush them into a paste […] Apply the aloe vera to the infested area. As the termites travel through the aloe vera, they will be coated and will suffocate, making it one of the more natural home remedies for termites”.
- Canola Oil: “Canola oil trap […] Wipe a small amount of canola oil across the infected area. Once a termite travels through the canola oil, it will suffocate because the oil will coat its outer shell, making respiration impossible” [178].

These untested home remedies listed above are easily recognized as resoundingly inadequate for subterranean termite control to anyone with training, but the danger is to the uninformed consumer. An examination of other boards also included misguided termite control recommendations such as “It is important to maintain cleanliness. Scraps of food, crumbs, accumulated dirt, excessive dust and moisture are the ones that create optimal conditions for the emergence and development of termites” [179]. Those knowledgeable of termite biology and behavior would understand that termites do not eat scraps of food and crumbs; however, this bit of advice is good for general pest management. Not all recommendations were incorrect. Some recommendations on Pinterest, such as keeping firewood stored away from the home or calling a professional, are good recommendations. The challenge lies in sorting credible recommendations from the misguided. 

## 10. Knowing Good Advice from Bad: A Challenge

The use of the Internet and social media is staggering. In 2021, 4.66 billion people, 59.5% of the world’s population, were active Internet users worldwide [180]. Google held 87.76% of the search engine market [181], and it is projected that 3.43 billion social media users will be active by 2023 [182]. Not all online information on the Internet is credible. It can be challenging to distinguish good information from bad for untrained personnel.

University extension systems are facing a new dilemma. Our content is evidence-based. Information shared is factual and often peer reviewed. However, traditional content delivery methods (i.e., extension circulars, bulletins, reviewed recommendations) do not have the reach that unreviewed recommendations on popular social media platforms do. The University of Florida is one of the larger extension systems in the U.S. For *all* of 2020, the University of Florida’s extension publication system had 17.5 million views for the *entire year* (Hagen, personal communication). Urban entomology extension publications averaged 1000–1500 views, with a few pulling in about 1800 (Oi, personal communication). By comparison, Pinterest had 459 million active users in the *fourth quarter* of 2020 alone [183]. The author of the board with 23 Home Remedies on Pinterest amassed an enviable 95,100 followers. The other boards I viewed reported followers ranging from 1100 to 1.3 million people for content developed by authors with little evidence of expertise in termite control and no apparent review process before posting. 

Perhaps even more formidable than Pinterest is YouTube, because YouTube does not just tell the user what to do, it can show a user how to do it. In 2020, an estimated 2.1 billion people used YouTube globally [184]. A search of YouTube for “how to kill termites naturally” resulted in videos that contained similar recommendations as those in Section 9.3, *Home Remedies*. For example, “How to kill termites and get rid of them forever” provided details on how to kill termites with salt, boric acid, essential oils, and Vaseline in 3 min and 40 s [185]. This single video listed 1.2 million views over 3 years (averaged to 400,000 views per year), which is about 400 times more views than a peer-reviewed urban pest management University of Florida extension publication. The Natural Cures channel lists 3.22 million subscribers.

*Why carry out your own termite control?* In a survey of 500 households, some 74% of homeowners reportedly carried out some kind of do-it-yourself pest control [186]. There are no specific studies on why consumers choose to carry out their own termite control or choose alternatives instead. However, there are related studies. In one survey of United Kingdom residents, DIY pest control was preferred over professional pest control except for wasps [187]. This study was primarily focused on wildlife. The speed of solving the problem was important to those who used professional pest control and do-it-yourself options [187]. 

Numerous studies have examined the parameters that influence the purchasing behaviors of DIY consumers [188] and the motivation to “do it yourself” [189,190]. Wolf and McQuitty [190] found that DIY behavior was driven by four factors: (1) economic benefit, (2) “a perceived lack of quality from available offerings, (3) a lack of product availability, and (4) the need for customization”. The immense reach of the Internet and social media has provided broad access to information on DIY projects which is generally beneficial for ideas on home decorating, fashion, food, or small home maintenance tasks where the risk of mistakes is small. The risk for an ineffective termite treatment can be high.

Pesticides carry extra concerns for consumers and may be an additional motivator for do-it-yourself options. Some non-pest management professionals distrust the pest control industry, are afraid of the pesticides being used, and seek “natural” alternatives to termite control. While there are no consumer attitude surveys specific to termite control, there is one survey about pest control in general. Four percent of consumers (N = 2027) in this survey indicated that they had a negative feeling toward the professional pest control industry, 38% were neutral, and 58% felt positively toward the industry [191]. While most consumers surveyed felt positively about the industry, there remains a disconnect in the data with consumer trust. Harridge-March [192] noted that trust is used as a shortcut in lieu of complex decision-making processes for decisions that carry risk. Additionally, people who are more trusting are less likely to see risk [192]. The large number of people who believe that home remedies will cure their termites probably do not truly understand the risk of termites to their homes.

The question of concern, why someone would elect to carry out their own termite control or use home remedies given the complexity of the task and risk of what is likely a high dollar investment, is complex. The simple answer is that most are unaware of the complicated nature of termite control, and it is easy to find YouTube videos that provide step-by-step guidance in about 3 min that make termite control seem easy to anyone wanting to do a treatment. “How-to” videos exist for U.S. EPA registered products and home remedies. They are free and available to users on demand. Compare the 3-min, step-by-step video to the training a pest management professional undergoes. In Florida, to become a certified operator in the category of wood-destroying organisms, someone would have to be mentored under another certified operator in the wood-destroying organisms category for 3 years and provide proof of performing 45 termite control services [193] (FS 482.132). A technician undertaking termite work would have to be trained for a minimum of 40 h before working independently and be supervised under a certified operator in the category of wood-destroying organisms. Other states have different, and sometimes more stringent, requirements. Further fueling the ease of DIY termite control is that the same products are available to both non-pest professionals and pest management professionals. Non-pest professionals can order it online and have it delivered to their home where it can be applied without the regulatory oversight that professional pest control works under. 

## 11. Concluding Thoughts

Alternative methods, for example, the use of nanoparticles [194,195], will continue to be researched as a first step toward evolving termite control methods. Peters et al. [195] attempted field colony elimination with fipronil-loaded silica nanocapsules without success. The authors stipulated that a better understanding of termite behavior was needed. Alternative methods must not only consider termite behavior and biology, products intended to confer structural protection must also consider (1) educating people, including those from other industries, about termite control, (2) building construction, and (3) modernizing product performance standards (i.e., regulatory requirements). 

For most people, the saying “your home is your number one investment” is true. According to the U.S. Census, a primary residence is the most highly valued asset [196]. Wealth is defined as assets minus debt, and 61.8% of households have built their wealth through equity invested in their primary residence. The median value of home equity was USD 118,000. The median value of a home in the U.S. between 2015 and 2019 was USD 217,000 [197], which means that many still have ~USD 100,000 to pay to a mortgage company or other lender. One is still responsible for paying the remaining mortgage regardless of any damage due to termites. To further place these figures in perspective, the median value of household wealth in the U.S. was USD 104,000, which includes households who do not own homes. Home equity accounted for 28.9% of household wealth. Homeownership is a critical path to building wealth. Given these data, proven termite control should be of utmost importance and a part of provisions for home maintenance that protect people’s number one investment.

It seems that we have traveled full-circle in termite control. As with the other categories of pest management, advances in research have developed effective products for control, but people’s behavior continues to be the most challenging aspect of protection against termites. In 1876, Hagen [11] observed that a large number of remedies were tried and found to be ineffective. In the 1800s, remedies were ineffective because the research that provided insight into understanding that termites live in highly organized colonies had not yet been conducted. We now better understand termite biology and behavior, enabling us to predict that home remedies will be ineffective compared to those products that meet a performance standard and claim structural protection. Regrettably, regardless of hundreds of research studies supporting U.S. EPA registered products, ineffective home remedies continue to proliferate with greater distribution through the Internet.

We have traveled full-circle in untested home remedies. In 1933, Shands [198] warned against “quack remedies”, adding:

“…Only a very few of the big corporations spending world of money in research work have been successful in developing a satisfactory treatment. Is it reasonable to supposed that one of these so-called experts could be so brainy or so lucky as to develop a successful treatment in a few days? There are literally hundreds of people who have had their homes treated…and still have them as badly as ever”.

The struggle to deter ineffective treatments while encouraging effective treatments is inextricably tied with effective communication strategies. Online bloggers are not regulated, nor do they have the same level of relationship with customers; thus, they may not feel the same level of responsibility for their content compared to a regulated company that will be responsible for damages that result from an improper treatment. It is clear that the Internet and social networking platforms can strongly influence consumers in termite control choices, particularly via electronic word of mouth (e-WOM) [199,200,201]. 

We have traveled full-circle with do-it-yourself efforts. We understand that the expert application of products is a combination of art and science. Pest management professionals must meet minimum training standards. In 1933, Fellman [202], a Terminix manager, wrote:

“It is absolutely impossible for anyone to successfully treat a termite infested structure by inexpensively squirting or otherwise applying a few gallons of some termiticide. A. comprehensive knowledge of the habits of termites, together with the proper equipment, and a chemical which has proved its efficiency against termites, are all necessary requirements. Even then, because of the human factor involved in the treatment work, perfect success cannot always be expected with the original application”.

The very first recommendations on termite control started with building construction and avoiding conducive conditions. In 1933, Shands [198] commented that:

“…we often find a tendency of the home owner to blame the architect or contractor this infestation. This may be unfair. When your house was built there was but little consideration of this pest. Your house was probably built along the established modern lines, with no consideration taken of termites. We do think that if this is not guarded against properly in the future you can certainly blame them for not taking the proper precautions”.

We have a better understanding of how building construction can contribute to termite protection, and this is incorporated into building codes globally. Nevertheless, working with building contractors who are tasked with implementing the architectural plan is critical. Efforts at termite control and their alternatives are an evolving process that necessarily involves working with people. Thus, while termite control methods will evolve, there will always be a need to educate a neverending stream of new consumers. 

## Figures and Tables

**Table 1:** Chronological list of chemicals tested as soil termiticides up to 1970. None of these chemicals are registered as termiticides today.

Chemicals Tested	Reference
Orthodichlorobenzene, trichlorobenzene, crude dichloropentane, crude diamyl phenol	[27]
Diphenylamine	[28]
DDT	[29]
Lead arsenate, sodium fluosilicate, cryolite phenothiazine, diphenylamine, pthalonitrile, trichlorobenzene, orthochlorobenzene, DDT creosote	[30]
DDT, dichlorodiphenyldichloroethane, methoxychlor, lindane, chlordane, pentachlorophenol, sodium pentachlorophenate, toxaphene, parathion	[31,32,33]
DDT, chlordane, toxaphene, heptachlor, lindane, aldrin, dieldrin, parathion, malathion, diazinon, pentachlorophenol, sodium pentachlorophenate, sodium arsenate	[34]
Aldrin, dieldrin, chlordane, DDT, heptachlor, lindane, and sodium arsenate	[35,36]

**Table 2:** Types of studies designed to improve termiticide efficacy.

Examples	Reference
Responses to natural products	[60]
Impact of soil type on termiticide efficacy	[61]
Efficacy of borates in soil	[62]
Termiticide persistence	[63,64]
Tunneling responses to termiticides of field compared to laboratory population	[65]
Termiticide distribution in different soils relative to the application equipment used (i.e., subslab injectors)	[66]
Foam applications to construction voids	[66,67]

## Data Availability

Review article references are in the reference section.
